# On Dysentery, Its Forms and Consequences

**Published:** 1835-04-01

**Authors:** 


### ON DYSENTERY, ITS FORMS AND CONSEQUENCES IN WARM CLIMATES, PARTICULARLY IN INDIA. By *James Annesley*, Esq. Chap. IV. Vol. II. Quarto.

In a former Number of this Journal (Vol. 15, p. 102,) we brought down the subjects of the second quarto volume of Mr. Annesley's work to the end of acute dysentery. It will now be proper to lay before our readers the substance of what this experienced writer and talented observer has said respecting "CHRONIC DYSENTERY and DIARRHŒA." These complaints are not of very unfrequent occurrence even in this country, and they are among the most common sequelæ of diseases contracted in the East, and presenting themselves to the practitioners of the West, when patients return to their native climes. They are therefore deserving of attention.

To Mr. Annesley chronic dysentery and chronic diarrhœa appear to depend on the same pathological state of the intestinal canal—differing only in degree—or perhaps in their seat. "In the former (chronic dysentery) the mucous coat and follicles of the small intestines seem to be chiefly affected;—in the latter (diarrhœa) the same textures of the large bowels are the seat of the disease." 239. The nature of the disease, he considers to be more doubtful than its seat. He is inclined, however, to view it "as the result of inflammatory action of a slow or chronic kind, occasioning organic changes in the structure of the part affected, consisting chiefly of enlargement and ulceration of the mucous follicles, with thickening and other lesions of the internal tunics of the bowel." We need not dwell on the symptoms of chronic dysentery. They are, on a small scale, those of the acute species, with the entire absence of some. The tormina are either entirely wanting, or only slight in degree, and the tenesmus is much less urgent.

"The stools are generally more or less serous, mucous, muco-purulent, and gelatinous, containing fluid, feculent matter, and substances varying in colour from a white, albuminous appearance, resembling the white of an egg, to a dark olive-green, or greenish black. Sometimes they are variegated or marbled, and occasionally one day seeming like chalk and water, and on another like a dark-coloured jelly, or the green fat of a turtle." 340.

There is little if any fever, especially in the morning; though an evening febrile movement is common in most chronic diseases.

*Chronic Dysentery* is generally the sequence of the acute form, or of repeated attacks of diarrhœa or cholera, as also of fevers and hepatitis. *Chronic diarrhœa* is, bonâ fide, but a milder degree of the other disease, and, in no essential respect, differing from it—unless as to the particular locality of the intestinal canal affected, if the doctrine of our author be admitted as strictly correct. Both diseases are often associated with chronic hepatitis, and this complication has been sufficiently noticed when we were examining the first volume of the work under review.

"Amongst these symptoms, a dark green appearance of the evacuations, indicating a morbid state of the bile; or a pale clay colour, shewing a torpid state of the liver, or obstruction of the biliary ducts; a dirty, watery, and offensive state of the stools; a pearly appearance of the eye; oppression or tightness at the epigastrium and lower part of the thorax; and sallow, muddy state of the countenance; with slight evening exacerbations of fever, and increased frequency of the calls to stool, are the most constant and prominent." 342.

The morbid secretions of the diseased liver are looked upon by our author as the cause of the diarrhœa or dysentery. Even the absence of bile is considered by Mr. A. as a source of irritation in the intestinal canal. When the disease terminates fatally, it is generally by ulceration and perforation of the intestinal tube. Sometimes the colon becomes constricted in consequence of spasmodic action long continued and accompanied by inflammation. This constriction causes accumulation and distention of the bowel above the stricture, with rupture of the gut, and extravasation of its contents. The functions of the kidneys and bladder are often disturbed in those cases. Simple and uncomplicated dysentery and diarrhœa are rarely met with in India:—they are generally complicated with hepatitis, splenitis, and other visceral affections, especially when they present themselves as the sequelæ of fevers. The causes of chronic dysentery and diarrhœa are altogether the same as of the acute forms of the disease. The appearances on dissection are somewhat different from those which we note after death from the acute form, and therefore may be worthy of record.

"The omentum is sometimes thickened, corrugated, drawn up to the colon, or to one side: it is not infrequently adherent to some part of the bowels, or to the brim of the pelvis, and occasionally to both. The stomach and small intestines are generally much distended with an offensive gas: more rarely the small intestines are irregularly constricted and thickened in their coats; and we have even found very extensive intus-susceptions in various parts of the ilium. With respect to their colour externally, they are generally very similar to that already described as belonging to the acute disease. Occasionally, firm and cellular adhesions are found connecting the external surface of the small intestines to the omentum, or to the cæcum or colon, or even one convolution of them to another.

The cæcum generally presents decided appearances of disease externally, but its internal structure is most extensively deranged: it is frequently distended with flatus, its coats thickened, its peritoneal surface covered in parts with coagulated lymph, and the cellular substance connecting it to the abdominal parietes inflamed, thickened, and easily lacerated. The colon is frequently distended in one part and contracted in another; but it sometimes is found very much enlarged and distended throughout, and devoid of its usual divisions into cells or compartments. Coagulable lymph is often seen on its surface, firmly uniting one part of it to another, and to the adjoining viscera, especially to the liver, stomach, spleen, &c. This is most frequently observed when ulcerations have nearly perforated all the tunics of the bowel, and the inflammation proceeded to its peritoneal covering.

Occasionally, when adhesions have taken place between adjoining parts of the peritoneum covering these viscera, or when the internal ulcerations have proceeded so far as to have induced peritonitis, and the patient has lived some time afterwards, the peritoneum presents appearances of chronic inflammation throughout a greater or less extent of surface, it being greatly thickened, more vascular, and the adhesions firm and organized.

The internal surface of the bowels generally presents the most constant and most extensive lesions in the forms of disease we are now considering. The coats of the small intestines are often tumid and thickened, especially the mucous and submucous tunics, with ulcerations in every stage of their progress. Accompanying this state, there are also observed, more or less, signs of inflammatory action, either in the seat of the ulcerations or in the spaces between them. The ulcers, in chronic diarrhœa, generally commence in the follicular glands, and are most numerous in the ilium, particularly in the lower parts of it. The mucous surface surrounding the ulcerations is often thickened, elevated, and of a deeper colour than natural. Sometimes the ulcers are small, numerous, and agglomerated in patches, conformably to the disposition of the follicles of the intestines: at other times, and in different parts of the bowel, the ulcers are large, distinct, few in number, and placed distantly from each other. Occasionally the surrounding texture is pale, and the edges of the ulcers thin and soft; frequently they are elevated upon thickened bases, and their edges prominent and rounded.

Ulceration is seldom met with in the small intestines, even in chronic diarrhœa, unattended with the characteristic symptoms of dysentery, without extensive ulceration being also present in the cæcum or sigmoid flexure of the colon. When the dysenteric symptoms have been present, the disease of the rectum, colon, and cæcum, is generally very manifest, and usually consists of ulcerations similar to those now enumerated, and to those described in the section on the appearances after the more active forms of the malady. Conjoined with ulceration, a contracted state of the bowels, particularly of the colon, at its left and sigmoid flexures, is generally present; and the parietes of the intestinal canal, sometimes of the small intestines, and almost always of the large bowels, are more or less thickened. Occasionally, the coats of the colon, rectum, and cæcum, are not only thickened and internally ulcerated, but also much indurated, and converted into a gristly or semi-cartilaginous state, and generally of a very dark hue, evidently owing to the long-continued irritation and inflammatory action kept up in the part, sometimes from the nature of the disease and peculiarity of constitution, and occasionally from inappropriate treatment, as shewn in some of the following cases.

The indurated and thickened state of the coats of the intestines is a very evident result of slow inflammatory action, especially as those states are generally either associated with ulcerations of a chronic kind, or with considerable contraction of the calibre of the bowel at the part thus changed in its organisation. Although not so lacerable as in those cases which have terminated fatally, the coats of the diseased intestines are generally more easily torn than in the healthy state, unless when they are of the gristly hardness already noticed.

Constriction of a part or parts of the colon, most frequently of the left arch, descending colon, and sigmoid flexure, are amongst the most constant appearances observed upon examinations after death from the chronic forms of the disease now before us. These constrictions may be few or they may be many—they are often of limited extent, resembling the ligature made by a cord, and frequently embrace a large portion of the bowel. They are generally accompanied with ulceration and thickening of the internal tunics of the intestine, but not uniformly so; and they usually present signs, either internally or externally, or both, of inflammatory action.

These strictures are, from their situation, beyond the reach of art, and little more can be done than to keep the contents of the bowels in a fluid state when we have reason to believe that they exist. But strictures also take place as a consequence of the various forms of dysentery and diarrhœa, between the sigmoid flexure of the colon and rectum, and in the rectum itself. It is chiefly to constrictions in this latter situation that attention has been directed by writers; and the idea that they are limited to the lower parts of the large bowel has been too generally entertained. A knowledge of the frequent occurrence of strictures in various parts of the colon, especially in its descending and sigmoid flexures, is of the utmost consequence in practice; and the frequent association of stricture of this bowel with that of the rectum is equally important, inasmuch as it teaches us not to confine our methods of cure to the rectum itself, but to extend them to the large bowels generally, as far as this end can be accomplished by means of gentle laxatives and emollient enemata." 349.

Mr. A. thinks that stricture of the rectum is not so common as is generally supposed. Among the Europeans in India, the colon was much more frequently affected. Contractions in the colon too often produce distention and even ulceration in the small intestines above. Several cases and dissections are given by our author, in illustration of the foregoing observations.

### TREATMENT.

Mr. A. properly remarks that, when the dysenteric symptoms following an acute attack consist chiefly of frequency of evacuations, without straining or tormina, the general health improving in the mean time, astringents should not be exhibited. The evacuations, he thinks, ought to be viewed as the efforts of Nature to relieve congested or sub-inflamed surfaces.

" When the motions are morbid, or attended with abdominal soreness, sense of heat, griping, tormina, tenesmus; if they be slimy, or at times sanguineous, and the patient complains much of thirst, and of fever, with restlessness at night,—the disease evidently possesses a character which must be removed by art, and which nature is generally incapable of counteracting, especially after an acute attack of disease. In cases of this description, the remains of inflammatory action should be dreaded as existing in complication with a morbid condition of the secretions; and judicious means should be resorted to, in order to remove both these states. Here local vascular depletions are necessary, especially if the patient have not been depleted early in the disease. If his strength has been lowered too far to admit of this measure, the employment of blisters to the abdomen, followed by a succession of hot poultices, and these by a thick flannel bandage, the warm-bath, and stimulating frictions upon the abdomen and lower limbs, will often prove serviceable." 369.

With the view of changing the morbid secretions, especially when the liver appears to be in fault, our author has been in the habit of giving " full doses of calomel and opium" at bed-time, and a gentle purgative in the morning—exhibiting the pil. hydrarg. with ipecacuan in the day, or rubbing in camphorated mercurial ointment on the hypochondria. At the same time, he uses emollient anodyne injections. After mercurials, the nitric acid with opium is recommended. In the latter stages of chronic dysentery, when there is much debility, infusion of cinchona, with rhubarb and tinct. opii, will be found beneficial.

"In those chronic cases which have been denominated '*white flux*,' from the muco-purulent and gleety appearance of the discharge, the muciparous glands of the large bowels are generally in a state of disease, and require the use of gentle tonics, combined with astringents, and alternated with purgatives and mercurial preparations. In the majority of these cases, the bile is either entirely obstructed, or it is secreted in insufficient quantity and quality. In order to restore the biliary secretion, and at the same time give energy to the relaxed mucous surface, of the colon, we have generally exhibited full doses of calomel at bed-time, with opium, and given the bitter aperient mixture in the morning, with advantage. Occasionally, we have also prescribed with benefit a pill, composed of aloes, calomel, and ipecacuanha, and directed an infusion of cinchona, rhubarb, and ginger to be employed, in the form of enema; or infusions of catechu, simarouba, columba, cinnamon, &c. in the same manner." 372.

In this country, where the complaint is chiefly met with among old East-Indians, great attention is necessary to keep the functions of the skin and the liver in good condition. Rest and farinaceous food are essential, and opiates, alternated with mild mercurials, will be found our sheet-anchor.

